# Hexaaqua­magnesium 5-[1-(carboxyl­ato­meth­yl)pyridin-1-ium-4-yl]tetra­zol-2-ide chloride dihydrate

**DOI:** 10.1107/S160053681105032X

**Published:** 2011-11-30

**Authors:** Yu Zhang

**Affiliations:** a Department of Physics, Southeast University, Nanjing 210096, People’s Republic of China

## Abstract

In the title compound, [Mg(H_2_O)_6_](C_8_H_6_N_5_O_2_)Cl·2H_2_O, the Mg^II^ ion is surrounded by six water mol­ecules, exhibiting a slightly distorted octa­hedral coordination. The pyridine and tetra­zole rings are nearly coplanar, forming a dihedral angle of 4.63 (3)°. The complex cations, zwitterionic organic anions, Cl^−^ anions and uncoordinated water mol­ecules are connected by O—H⋯O, O—H⋯N and O—H⋯Cl hydrogen bonds, leading to the formation of a three-dimensional network.

## Related literature

For related tetra­zole derivatives, see: Fu *et al.* (2009 ▶, 2010 ▶).
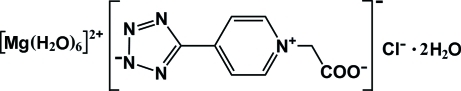


## Experimental

### 

#### Crystal data

[Mg(H_2_O)_6_](C_8_H_6_N_5_O_2_)Cl·2H_2_O
*M* *_r_* = 408.07
Monoclinic, 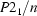

*a* = 8.1627 (16) Å
*b* = 12.896 (3) Å
*c* = 17.435 (4) Å
β = 96.85 (3)°
*V* = 1822.3 (7) Å^3^
*Z* = 4
Mo *K*α radiation
μ = 0.30 mm^−1^
*T* = 298 K
0.40 × 0.30 × 0.20 mm

#### Data collection

Rigaku Mercury2 diffractometer
Absorption correction: multi-scan (*CrystalClear*; Rigaku, 2005 ▶) *T*_min_ = 0.89, *T*_max_ = 1.00
18612 measured reflections
4172 independent reflections
3261 reflections with *I* > 2σ(*I*)
*R*_int_ = 0.044

#### Refinement

*R*[*F* ^2^ > 2σ(*F* ^2^)] = 0.061
*wR*(*F* ^2^) = 0.181
*S* = 1.10
4172 reflections
226 parameters
16 restraints
H-atom parameters constrained
Δρ_max_ = 1.00 e Å^−3^
Δρ_min_ = −0.41 e Å^−3^

### 

Data collection: *CrystalClear* (Rigaku, 2005 ▶); cell refinement: *CrystalClear*; data reduction: *CrystalClear*; program(s) used to solve structure: *SHELXS97* (Sheldrick, 2008 ▶); program(s) used to refine structure: *SHELXL97* (Sheldrick, 2008 ▶); molecular graphics: *XP* in *SHELXTL* (Sheldrick, 2008 ▶) and *DIAMOND* (Brandenburg, 1999 ▶); software used to prepare material for publication: *SHELXTL*.

## Supplementary Material

Crystal structure: contains datablock(s) I, global. DOI: 10.1107/S160053681105032X/hy2489sup1.cif

Structure factors: contains datablock(s) I. DOI: 10.1107/S160053681105032X/hy2489Isup2.hkl

Additional supplementary materials:  crystallographic information; 3D view; checkCIF report

## Figures and Tables

**Table 1:** Hydrogen-bond geometry (Å, °)

*D*—H⋯*A*	*D*—H	H⋯*A*	*D*⋯*A*	*D*—H⋯*A*
O1*W*—H1*WA*⋯N1^i^	0.82	2.07	2.883 (5)	174
O1*W*—H1*WB*⋯Cl1	0.82	2.36	3.180 (3)	173
O2*W*—H2*WA*⋯N3^ii^	0.82	2.07	2.885 (5)	178
O2*W*—H2*WB*⋯O1^iii^	0.82	1.99	2.793 (4)	167
O3*W*—H3*WA*⋯O1	0.82	1.91	2.722 (4)	170
O3*W*—H3*WB*⋯O7*W*^iv^	0.82	1.95	2.748 (4)	165
O4*W*—H4*WA*⋯O2^v^	0.82	1.94	2.738 (4)	164
O4*W*—H4*WB*⋯O8*W*^vi^	0.82	1.98	2.794 (4)	174
O5*W*—H5*WA*⋯Cl1^vii^	0.82	2.34	3.162 (3)	174
O5*W*—H5*WB*⋯O2	0.82	1.91	2.715 (4)	169
O6*W*—H6*WA*⋯O8*W*^ii^	0.82	1.97	2.763 (5)	164
O6*W*—H6*WB*⋯O7*W*^v^	0.82	1.86	2.678 (4)	178
O7*W*—H7*WA*⋯N2^viii^	0.82	1.94	2.748 (5)	169
O7*W*—H7*WB*⋯Cl1^vii^	0.82	2.29	3.106 (3)	170
O8*W*—H8*WA*⋯Cl1^iv^	0.91	2.26	3.109 (4)	156
O8*W*—H8*WB*⋯N4	0.82	2.00	2.822 (5)	179

Symmetry codes: (i) 
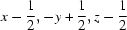
; (ii) 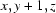; (iii) 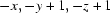; (iv) 
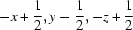
; (v) 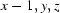; (vi) 
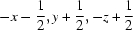
; (vii) 
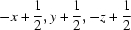
; (viii) 
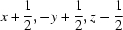
.

**Fig. 1.**
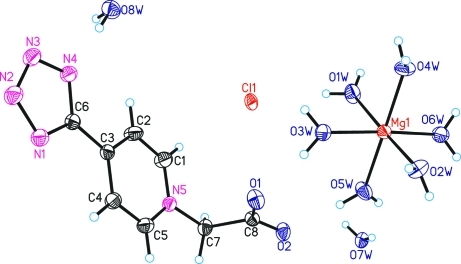
Molecular structure of the title compound. Displacement ellipsoids are drawn at the 30% probability level.

**Fig. 2.**
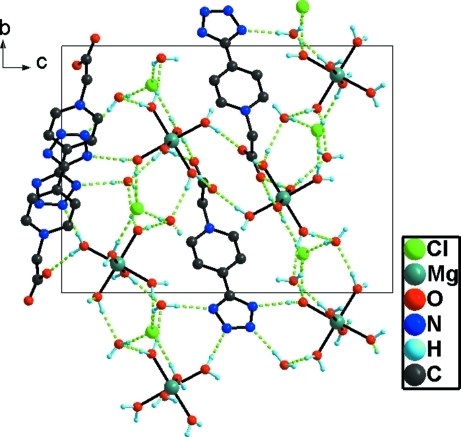
The crystal packing of the title compound, showing the three-dimensional hydrogen-bonded network. H atoms not involved in hydrogen bonds (dashed line) have been omitted for clarity.

## supplementary crystallographic information

### Comment

Molecule-based compounds have attracted more attention as phase transition dielectric materials for their applications in micro-electronics and memory storage. With the purpose of obtaining phase transition crystals of tetrazole compounds, the interactions of tetrazoles with various metal ions have been studied and a series of new materials have been elaborated (Fu *et al.*, 2010). In this paper, we describe the crystal structure of the title compound.

In the title compound, the asymmetric unit consists of one zwitterionic 5-[1-(carboxylatomethyl)pyridinium-4-yl]tetrazol-2-ide anion, one [Mg(H_2_O)_6_]^2+^ cation, one Cl^-^ anion and two uncoordinated water molecules. The Mg^II^ ion is surrounded by six water molecules, exhibiting a slightly distorted octahedral coordination. Mg—O bond distances range from 2.041 (3) to 2.092 (3)Å [mean value 2.059 (3)Å]. In the zwitterionic organic anion, the pyridine and tetrazole rings are nearly coplanar, only twisted from each other by a dihedral angle of 4.63 (3)°. The geometric parameters of the tetrazole rings are comparable to those in related molecules (Fu *et al.*, 2009). In crystal, the complex cations, and Cl^-^ anions are linked through O—H···Cl hydrogen bonds into a sheet parallel to (0 0 1). The sheets are linked by the organic anions and water molecules through O—H···N and O—H···O hydrogen bonds into a three-dimensional network (Table 1 and Fig. 2).

### Experimental

MgCl_2_.6H_2_O (2 mmol) and 1-(carboxymethyl)-4-(2H-tetrazol-5-yl)pyridinium (2 mmol) were dissolved in a 70% methanol aqueous solution, and then 2 ml HCl was added. Single crystals suitable for X-ray diffraction analysis were obtained by slow evaporation of the solution at room temperature after two weeks.

### Refinement

H atoms attached to C atoms were positioned geometrically and treated as riding, with C—H = 0.93 (aromatic) and 0.97 (methylene) Å and with *U*_iso_(H) = 1.2*U*_eq_(C). H atoms bonded to O atoms were located in difference Fourier maps and restrained with H—O = 0.820 (2)Å. In the last stage of refinements they were treated as riding on the O atoms with *U*_iso_(H) = 1.5*U*_eq_(O).

### Crystal data

**Table:**

[Mg(H_2_O)_6_](C_8_H_6_N_5_O_2_)Cl·2H_2_O	*F*(000) = 856
*M**_r_* = 408.07	*D*_x_ = 1.487 Mg m^−^^3^
Monoclinic, *P*2_1_/*n*	Mo *K*α radiation, λ = 0.71073 Å
Hall symbol: -P 2yn	Cell parameters from 4172 reflections
*a* = 8.1627 (16) Å	θ = 3.1–27.5°
*b* = 12.896 (3) Å	µ = 0.30 mm^−^^1^
*c* = 17.435 (4) Å	*T* = 298 K
β = 96.85 (3)°	Block, colourless
*V* = 1822.3 (7) Å^3^	0.40 × 0.30 × 0.20 mm
*Z* = 4	

### Data collection

**Table:**

Rigaku Mercury2 diffractometer	4172 independent reflections
Radiation source: fine-focus sealed tube	3261 reflections with *I* > 2σ(*I*)
graphite	*R*_int_ = 0.044
Detector resolution: 13.6612 pixels mm^-1^	θ_max_ = 27.5°, θ_min_ = 3.1°
profile data from φ scans	*h* = −10→10
Absorption correction: multi-scan (*CrystalClear*; Rigaku, 2005)	*k* = −16→16
*T*_min_ = 0.89, *T*_max_ = 1.00	*l* = −22→22
18612 measured reflections	

### Refinement

**Table:**

Refinement on *F*^2^	Primary atom site location: structure-invariant direct methods
Least-squares matrix: full	Secondary atom site location: difference Fourier map
*R*[*F*^2^ > 2σ(*F*^2^)] = 0.061	Hydrogen site location: inferred from neighbouring sites
*wR*(*F*^2^) = 0.181	H-atom parameters constrained
*S* = 1.10	*w* = 1/[σ^2^(*F*_o_^2^) + (0.0828*P*)^2^ + 2.4078*P*] where *P* = (*F*_o_^2^ + 2*F*_c_^2^)/3
4172 reflections	(Δ/σ)_max_ < 0.001
226 parameters	Δρ_max_ = 1.00 e Å^−^^3^
16 restraints	Δρ_min_ = −0.41 e Å^−^^3^

### Fractional atomic coordinates and isotropic or equivalent isotropic displacement parameters (Å^2^)

**Table:**

	*x*	*y*	*z*	*U*_iso_*/*U*_eq_	
Cl1	0.17139 (12)	0.34162 (8)	0.22732 (6)	0.0364 (3)	
Mg1	−0.08699 (16)	0.61711 (10)	0.33130 (7)	0.0310 (3)	
O1	0.2675 (4)	0.4216 (2)	0.45454 (18)	0.0442 (7)	
N1	0.2557 (4)	−0.0553 (3)	0.5764 (2)	0.0394 (8)	
O1W	−0.0632 (4)	0.5384 (2)	0.22830 (17)	0.0502 (8)	
H1WA	−0.1161	0.5389	0.1852	0.075*	
H1WB	−0.0078	0.4860	0.2245	0.075*	
C1	0.3765 (6)	0.2067 (3)	0.3919 (2)	0.0445 (10)	
H1A	0.3625	0.2297	0.3410	0.053*	
O2	0.4243 (4)	0.5313 (2)	0.39940 (19)	0.0429 (7)	
O2W	−0.1241 (4)	0.6896 (2)	0.43319 (17)	0.0453 (7)	
H2WA	−0.0667	0.7378	0.4515	0.068*	
H2WB	−0.1520	0.6576	0.4702	0.068*	
N2	0.1572 (5)	−0.1383 (3)	0.5687 (2)	0.0442 (9)	
C2	0.3020 (6)	0.1176 (3)	0.4109 (2)	0.0430 (10)	
H2A	0.2376	0.0802	0.3729	0.052*	
O3W	−0.0387 (4)	0.4836 (2)	0.39268 (17)	0.0430 (7)	
H3WA	0.0506	0.4679	0.4165	0.065*	
H3WB	−0.0725	0.4260	0.3784	0.065*	
C3	0.3210 (5)	0.0823 (3)	0.4857 (2)	0.0303 (8)	
N3	0.0837 (5)	−0.1434 (3)	0.4978 (2)	0.0408 (8)	
O4W	−0.3332 (4)	0.5816 (3)	0.31010 (18)	0.0459 (8)	
H4WA	−0.3908	0.5627	0.3429	0.069*	
H4WB	−0.3894	0.5449	0.2788	0.069*	
N4	0.1325 (4)	−0.0644 (3)	0.45715 (19)	0.0363 (8)	
C4	0.4211 (6)	0.1390 (3)	0.5397 (2)	0.0393 (9)	
H4A	0.4396	0.1161	0.5906	0.047*	
O5W	0.1614 (4)	0.6522 (2)	0.3482 (2)	0.0507 (8)	
H5WA	0.2015	0.7041	0.3308	0.076*	
H5WB	0.2369	0.6165	0.3694	0.076*	
N5	0.4697 (4)	0.2615 (2)	0.44566 (19)	0.0327 (7)	
C5	0.4932 (5)	0.2285 (3)	0.5186 (2)	0.0402 (9)	
H5A	0.5592	0.2668	0.5555	0.048*	
O6W	−0.1197 (4)	0.7529 (2)	0.27062 (19)	0.0480 (8)	
H6WA	−0.0840	0.8085	0.2884	0.072*	
H6WB	−0.2026	0.7635	0.2401	0.072*	
C6	0.2380 (5)	−0.0120 (3)	0.5067 (2)	0.0316 (8)	
C7	0.5290 (5)	0.3640 (3)	0.4247 (3)	0.0372 (9)	
H7A	0.5630	0.3611	0.3733	0.045*	
H7B	0.6241	0.3835	0.4605	0.045*	
O7W	0.6092 (4)	0.7924 (2)	0.17261 (18)	0.0480 (8)	
H7WA	0.6127	0.7428	0.1434	0.072*	
H7WB	0.5361	0.7978	0.2008	0.072*	
C8	0.3928 (5)	0.4459 (3)	0.4266 (2)	0.0322 (8)	
O8W	0.0012 (4)	−0.0492 (3)	0.30034 (18)	0.0519 (8)	
H8WA	0.0898	−0.0721	0.2784	0.078*	
H8WB	0.0403	−0.0529	0.3458	0.078*	

### Atomic displacement parameters (Å^2^)

**Table:**

	*U*^11^	*U*^22^	*U*^33^	*U*^12^	*U*^13^	*U*^23^
Cl1	0.0352 (5)	0.0379 (5)	0.0369 (5)	0.0131 (4)	0.0081 (4)	−0.0006 (4)
Mg1	0.0294 (7)	0.0299 (7)	0.0333 (7)	−0.0015 (5)	0.0025 (5)	0.0014 (5)
O1	0.0342 (15)	0.0489 (18)	0.0517 (18)	0.0035 (13)	0.0143 (13)	0.0126 (14)
N1	0.044 (2)	0.0372 (19)	0.0364 (18)	−0.0053 (15)	0.0004 (15)	0.0035 (14)
O1W	0.062 (2)	0.054 (2)	0.0343 (16)	0.0199 (16)	0.0024 (14)	−0.0062 (14)
C1	0.057 (3)	0.043 (2)	0.032 (2)	−0.012 (2)	−0.0015 (18)	0.0056 (18)
O2	0.0384 (16)	0.0329 (15)	0.0584 (19)	0.0015 (12)	0.0101 (13)	0.0107 (13)
O2W	0.0570 (19)	0.0406 (16)	0.0406 (16)	−0.0135 (14)	0.0151 (14)	−0.0071 (13)
N2	0.051 (2)	0.038 (2)	0.042 (2)	−0.0098 (16)	0.0023 (16)	0.0069 (15)
C2	0.049 (3)	0.045 (2)	0.033 (2)	−0.015 (2)	−0.0036 (18)	−0.0001 (17)
O3W	0.0451 (17)	0.0327 (15)	0.0486 (17)	−0.0014 (13)	−0.0055 (13)	0.0063 (13)
C3	0.0301 (18)	0.0289 (18)	0.0317 (19)	0.0029 (15)	0.0032 (14)	−0.0013 (15)
N3	0.044 (2)	0.0339 (18)	0.044 (2)	−0.0066 (15)	0.0054 (15)	0.0004 (15)
O4W	0.0316 (15)	0.0569 (19)	0.0494 (18)	−0.0115 (14)	0.0062 (13)	−0.0078 (15)
N4	0.0379 (18)	0.0347 (18)	0.0358 (18)	−0.0045 (14)	0.0023 (14)	0.0002 (14)
C4	0.052 (2)	0.034 (2)	0.0307 (19)	−0.0030 (18)	−0.0027 (17)	0.0027 (16)
O5W	0.0308 (15)	0.0462 (18)	0.074 (2)	−0.0025 (13)	0.0014 (14)	0.0215 (16)
N5	0.0304 (16)	0.0283 (16)	0.0396 (18)	0.0000 (13)	0.0048 (13)	0.0014 (13)
C5	0.045 (2)	0.038 (2)	0.036 (2)	−0.0046 (18)	−0.0032 (17)	−0.0014 (17)
O6W	0.0470 (17)	0.0367 (16)	0.0568 (19)	−0.0057 (14)	−0.0086 (14)	0.0105 (14)
C6	0.0322 (19)	0.0301 (19)	0.0325 (19)	0.0012 (15)	0.0041 (15)	−0.0008 (15)
C7	0.033 (2)	0.031 (2)	0.049 (2)	−0.0010 (16)	0.0107 (17)	0.0038 (17)
O7W	0.0518 (19)	0.0393 (17)	0.0522 (19)	0.0089 (14)	0.0036 (14)	−0.0070 (14)
C8	0.0312 (19)	0.033 (2)	0.0318 (19)	0.0005 (15)	0.0030 (15)	0.0020 (15)
O8W	0.058 (2)	0.054 (2)	0.0413 (17)	0.0037 (16)	−0.0047 (15)	−0.0012 (14)

### Geometric parameters (Å, °)

**Table:**

Mg1—O3W	2.041 (3)	C3—C4	1.380 (6)
Mg1—O6W	2.047 (3)	C3—C6	1.460 (5)
Mg1—O4W	2.052 (3)	N3—N4	1.330 (5)
Mg1—O2W	2.061 (3)	O4W—H4WA	0.8201
Mg1—O5W	2.064 (3)	O4W—H4WB	0.8201
Mg1—O1W	2.092 (3)	N4—C6	1.329 (5)
O1—C8	1.225 (5)	C4—C5	1.367 (6)
N1—C6	1.329 (5)	C4—H4A	0.9300
N1—N2	1.335 (5)	O5W—H5WA	0.8202
O1W—H1WA	0.8203	O5W—H5WB	0.8202
O1W—H1WB	0.8202	N5—C5	1.334 (5)
C1—N5	1.337 (5)	N5—C7	1.469 (5)
C1—C2	1.359 (6)	C5—H5A	0.9300
C1—H1A	0.9300	O6W—H6WA	0.8203
O2—C8	1.238 (5)	O6W—H6WB	0.8203
O2W—H2WA	0.8203	C7—C8	1.536 (5)
O2W—H2WB	0.8202	C7—H7A	0.9700
N2—N3	1.311 (5)	C7—H7B	0.9700
C2—C3	1.372 (6)	O7W—H7WA	0.8201
C2—H2A	0.9300	O7W—H7WB	0.8202
O3W—H3WA	0.8202	O8W—H8WA	0.9070
O3W—H3WB	0.8203	O8W—H8WB	0.8201
			
O3W—Mg1—O6W	176.38 (14)	N2—N3—N4	109.3 (3)
O3W—Mg1—O4W	91.72 (14)	Mg1—O4W—H4WA	124.9
O6W—Mg1—O4W	91.89 (14)	Mg1—O4W—H4WB	135.0
O3W—Mg1—O2W	88.26 (13)	H4WA—O4W—H4WB	88.3
O6W—Mg1—O2W	91.94 (14)	C6—N4—N3	104.8 (3)
O4W—Mg1—O2W	90.85 (14)	C5—C4—C3	120.2 (4)
O3W—Mg1—O5W	89.14 (13)	C5—C4—H4A	119.9
O6W—Mg1—O5W	87.24 (13)	C3—C4—H4A	119.9
O4W—Mg1—O5W	177.82 (15)	Mg1—O5W—H5WA	123.7
O2W—Mg1—O5W	91.18 (15)	Mg1—O5W—H5WB	127.9
O3W—Mg1—O1W	90.50 (14)	H5WA—O5W—H5WB	108.3
O6W—Mg1—O1W	89.51 (14)	C5—N5—C1	120.3 (4)
O4W—Mg1—O1W	85.77 (14)	C5—N5—C7	120.7 (3)
O2W—Mg1—O1W	176.36 (15)	C1—N5—C7	118.6 (3)
O5W—Mg1—O1W	92.22 (15)	N5—C5—C4	120.6 (4)
C6—N1—N2	104.3 (3)	N5—C5—H5A	119.7
Mg1—O1W—H1WA	133.6	C4—C5—H5A	119.7
Mg1—O1W—H1WB	125.3	Mg1—O6W—H6WA	122.2
H1WA—O1W—H1WB	99.7	Mg1—O6W—H6WB	121.5
N5—C1—C2	120.7 (4)	H6WA—O6W—H6WB	109.2
N5—C1—H1A	119.7	N4—C6—N1	111.8 (4)
C2—C1—H1A	119.7	N4—C6—C3	122.9 (3)
Mg1—O2W—H2WA	122.8	N1—C6—C3	125.2 (3)
Mg1—O2W—H2WB	122.2	N5—C7—C8	110.6 (3)
H2WA—O2W—H2WB	106.0	N5—C7—H7A	109.5
N3—N2—N1	109.7 (3)	C8—C7—H7A	109.5
C1—C2—C3	120.6 (4)	N5—C7—H7B	109.5
C1—C2—H2A	119.7	C8—C7—H7B	109.5
C3—C2—H2A	119.7	H7A—C7—H7B	108.1
Mg1—O3W—H3WA	125.2	H7WA—O7W—H7WB	121.4
Mg1—O3W—H3WB	124.6	O1—C8—O2	127.0 (4)
H3WA—O3W—H3WB	100.2	O1—C8—C7	118.2 (3)
C2—C3—C4	117.6 (4)	O2—C8—C7	114.8 (3)
C2—C3—C6	120.8 (3)	H8WA—O8W—H8WB	98.7
C4—C3—C6	121.6 (3)		
			
C6—N1—N2—N3	−0.4 (5)	N3—N4—C6—N1	−0.3 (5)
N5—C1—C2—C3	0.1 (7)	N3—N4—C6—C3	−179.5 (4)
C1—C2—C3—C4	−1.7 (7)	N2—N1—C6—N4	0.4 (5)
C1—C2—C3—C6	178.5 (4)	N2—N1—C6—C3	179.7 (4)
N1—N2—N3—N4	0.2 (5)	C2—C3—C6—N4	−4.7 (6)
N2—N3—N4—C6	0.0 (5)	C4—C3—C6—N4	175.5 (4)
C2—C3—C4—C5	2.1 (6)	C2—C3—C6—N1	176.2 (4)
C6—C3—C4—C5	−178.1 (4)	C4—C3—C6—N1	−3.7 (6)
C2—C1—N5—C5	1.1 (7)	C5—N5—C7—C8	−92.9 (4)
C2—C1—N5—C7	−171.8 (4)	C1—N5—C7—C8	80.0 (5)
C1—N5—C5—C4	−0.7 (6)	N5—C7—C8—O1	10.1 (5)
C7—N5—C5—C4	172.1 (4)	N5—C7—C8—O2	−170.4 (3)
C3—C4—C5—N5	−1.0 (7)		

### Hydrogen-bond geometry (Å, °)

**Table:**

*D*—H···*A*	*D*—H	H···*A*	*D*···*A*	*D*—H···*A*
O1W—H1WA···N1^i^	0.82	2.07	2.883 (5)	174
O1W—H1WB···Cl1	0.82	2.36	3.180 (3)	173
O2W—H2WA···N3^ii^	0.82	2.07	2.885 (5)	178
O2W—H2WB···O1^iii^	0.82	1.99	2.793 (4)	167
O3W—H3WA···O1	0.82	1.91	2.722 (4)	170
O3W—H3WB···O7W^iv^	0.82	1.95	2.748 (4)	165
O4W—H4WA···O2^v^	0.82	1.94	2.738 (4)	164
O4W—H4WB···O8W^vi^	0.82	1.98	2.794 (4)	174
O5W—H5WA···Cl1^vii^	0.82	2.34	3.162 (3)	174
O5W—H5WB···O2	0.82	1.91	2.715 (4)	169
O6W—H6WA···O8W^ii^	0.82	1.97	2.763 (5)	164
O6W—H6WB···O7W^v^	0.82	1.86	2.678 (4)	178
O7W—H7WA···N2^viii^	0.82	1.94	2.748 (5)	169
O7W—H7WB···Cl1^vii^	0.82	2.29	3.106 (3)	170
O8W—H8WA···Cl1^iv^	0.91	2.26	3.109 (4)	156
O8W—H8WB···N4	0.82	2.00	2.822 (5)	179

Symmetry codes: (i) *x*−1/2, −*y*+1/2, *z*−1/2; (ii) *x*, *y*+1, *z*; (iii) −*x*, −*y*+1, −*z*+1; (iv) −*x*+1/2, *y*−1/2, −*z*+1/2; (v) *x*−1, *y*, *z*; (vi) −*x*−1/2, *y*+1/2, −*z*+1/2; (vii) −*x*+1/2, *y*+1/2, −*z*+1/2; (viii) *x*+1/2, −*y*+1/2, *z*−1/2.
